# Early detection of metabolic changes in drug-induced steatosis using metabolomics approaches†

**DOI:** 10.1039/d0ra06577c

**Published:** 2020-11-11

**Authors:** Helena Y Yong, Gerald Larrouy-Maumus, Mire Zloh, Rosemary Smyth, Rayan Ataya, Christopher M. Benton, Michael R. Munday

**Affiliations:** Department of Pharmaceutical and Biological Chemistry, University of London UK h.yong@ucl.ac.uk; MRC Centre for Molecular Bacteriology and Infection, Department of Life Science, Faculty of Natural Sciences, Imperial College London UK; Agilent Technologies Ltd Cheshire UK

## Abstract

Steatosis is the accumulation of triglycerides in hepatic cells wherein fats exceed 5% of the entire liver weight. Although steatotic liver damage is reversible due to the liver's regenerative capability, protracted damage often and typically leads to irreversible conditions such as cirrhosis and hepatocellular carcinoma (HCC). Therefore, early steatotic detection is critical for preventing progression to advanced liver diseases. This also becomes particularly important given the higher prevalence of drug usage, as drugs are a frequent cause of liver damage. Currently, the recommendation to diagnose steatosis is using liver enzymes and performing a liver biopsy. Liver biopsy remains the gold standard method of detection, but the procedure is invasive and an unreliable diagnostic tool. Non-invasive, specific and sensitive diagnostic solutions such as biomarkers are therefore needed for the early detection of steatosis. Our aim is to identify changes in urinary metabolites in tetracycline-induced hepatic steatotic rats at different stages of the diseases using metabolomic-based techniques. Sprague Dawley male rats are treated by intraperitoneal injection (I.P.) with either 62.5 mg kg^−1^ or 125 mg kg^−1^ tetracycline, an antibiotic previously known to induce steatosis. We analyse the metabolic profile of the urinary tetracycline induced hepatic steatotic rats using ^1^H nuclear magnetic resonance (NMR), 2D ^1^H–^1^H TOCSY (total correlation spectroscopy) and electrospray liquid chromatography-mass spectrometry (ESI-LC-MS/MS) based metabolomics. The combined analysis of haematoxylin & eosin (H&E), oil red O (ORO) and direct measurement of triglyceride content in the liver tissues of the control samples against 125 mg kg^−1^ and 62.5 mg kg^−1^ treated samples, reveals that 125 mg kg^−1^ tetracycline exposure potentially induces steatosis. The combination of ^1^H NMR, 2D ^1^H–^1^H TOCSY and ESI-LC-MS/MS alongside multivariate statistical analysis, detected a total of 6 urinary metabolites changes, across 6 metabolic pathways. Furthermore, lysine concentration correlates with liver damage as tetracycline dose concentration increases, whilst both H&E and ORO fail to detect hepatocellular damage at the lowest dose concentration. We conclude that the combination of ^1^H NMR and ESI-LC-MS/MS suggests that these are suitable platforms for studying the pathogenesis of steatosis development, prior to morphological alterations observed in staining techniques and offer a more detailed description of the severity of the steatotic disease.

## Abstract 2

Urinary metabolic profiling of tetracycline induced hepatic steatotic rats were investigated using ^1^H nuclear magnetic resonance, 2D ^1^H–^1^H total correlation spectroscopy and electrospray liquid chromatography-mass spectrometry based metabolomics.

## Graphical abstract

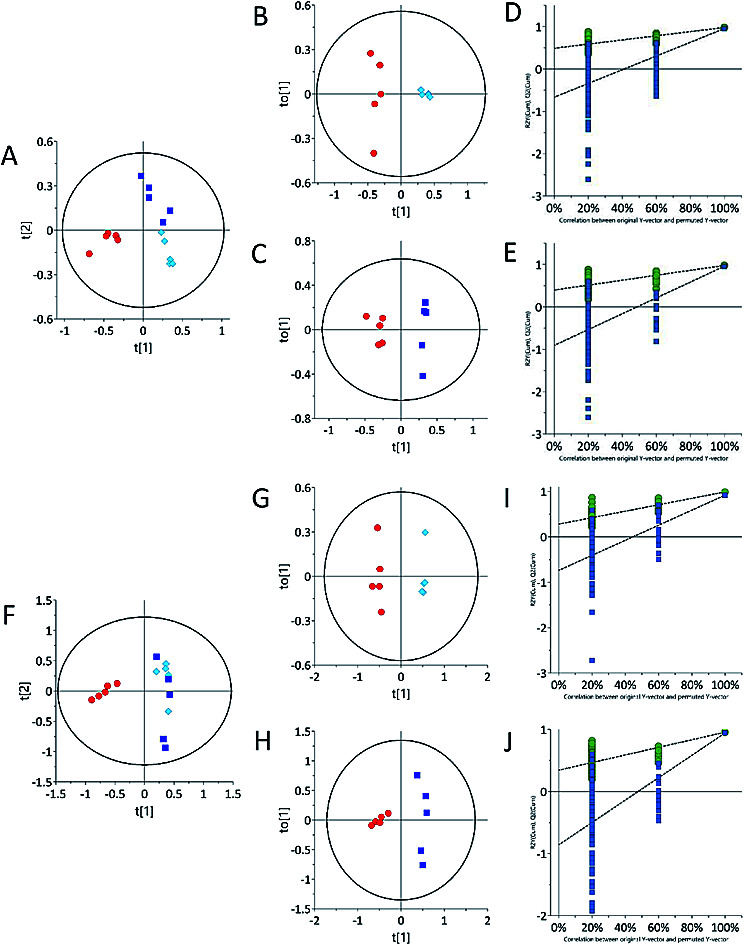

## Introduction

Non-alcoholic fatty liver disease (NAFLD) has become one of the leading causes of chronic liver disease globally.^1–3^ NAFLD represents a spectrum of diseases ranging from simple steatosis progressing to nonalcoholic steatohepatitis (NASH), fibrosis, cirrhosis and hepatocellular carcinoma (HCC).^4,5^

Steatosis, also known as fatty liver, is caused by hepatic cells retaining abnormal levels of triglycerides, wherein fats exceed 5% of the entire liver weight.^2,5–8^ Whilst damage here is typically reversible given the liver's high regenerative capability,^9^ early detection remains critical for preventing progression towards irreversible conditions, which include cirrhosis and HCC.^4,5,10^

Abnormal enzyme liver function tests (serum aminotransferase enzymes) and non-invasive methods (ultrasound or ^1^H-magnetic-resonance spectroscopy) are used to diagnose liver disease such as steatosis.^11^ Steatosis is usually diagnosed by the measurement of gamma-glutamyl transferase (GGT) and alanine aminotransferase (ALT)^12^ and in addition ultrasonography. Ultrasound is commonly used in detecting hepatic steatosis and can also provide grading of the hepatic fat content based on the visual assessment.^13^ However, it is difficult to detect small changes in hepatic fat content with time and therefore not useful for early or mild steatosis.^14^ Once hepatic steatosis is diagnosed the difficultly then is to measure the degree of severity of the disease.^12,15^ Therefore, liver biopsy remains the gold standard for detection and prognosis but the procedure is invasive and prone to sampling error.^16^ Furthermore, due to the asymptomatic nature of the condition there is a need for a more sensitive, specific and less invasive form of detection.

The use of drugs is a frequent cause of steatosis,^17–19^ ranging across multiple therapeutic classes including antiarrhythmic agents, chemotherapeutics, and tetracyclines.^6,20–22^ High doses and prolonged use of tetracycline, for treatment of a range of bacterial infections,^23^ has been shown to cause steatosis and liver damage in animals^23–26^ clinically similar to the human pathology of liver steatosis assessed by histological staining.^27–29^ Animal studies play an important role in studying and understanding the pathogenic processes of the human disease.^30^ Tetracycline-induced steatosis animal models have been used to elucidate the mechanism underlying the pathogenesis of hepatic steatosis and search for diagnostic biomarkers.^19^ Various cellular mechanisms are indicated as contributing to steatotic onset, including impairment of the mitochondrial enzymes, impaired fatty acid oxidation and peroxisome proliferator-activated receptor (PPARα) pathways causing a decrease in catabolism of fatty acids, reduced synthesis and secretion of very-low-density lipoprotein (VLDL), and a potential increase in lipogenesis.^6,20,21,25,31,32^ Researchers have investigated altered chemical pathways^27,33–36^ drug-induced steatosis through metabolite changes in serum, urine and hepatic tissues from animals and patients.^19,22,24,25,37–41^ These studies have identified several metabolites associated with various pathways related to the pathogenesis of drug induced steatosis. However, these studies have limitations identifying changes in biomarkers in the early development and progression of the disease.^35^ Therefore, more sensitive and specific biomarkers are required to diagnose the progression or severity of drug induced liver steatosis.^42^

Here, we investigate urinary metabolites from tetracycline-induced hepatic steatotic rats, using both ^1^H nuclear magnetic resonance (NMR) and liquid chromatography-mass spectrometry (ESI-LC-MS/MS) based metabolomics. We find a number of potential biomarkers whose change in concentration correlates with the progression of steatosis, producing leads towards the most significant biochemical pathways. We also find that haematoxylin & eosin (H&E) and oil red O (ORO) staining is less sensitive in the detection of the disease at lower dose levels of drug compared to metabolomics and may therefore be less reliable in tracking steatotic severity.

## Experimental details

### Materials and reagents

Monobasic sodium phosphate (NaH_2_PO_4_), dibasic sodium phosphate (Na_2_HPO_4_), sodium chloride (NaCl), ethanol, potassium hydroxide (KOH), magnesium chloride (MgCl_2_), methanol and formalin fixative were purchased from Sigma-Aldrich Co. Ltd, Gillingham, Dorset, UK. Tetracycline hydrochloride was purchased from Fluka Chemicals, Gillingham, Dorset, UK. Trimethylsilyl propionate (TSP), and deuterated water (D_2_O) were purchased from Cambridge Isotopes Laboratories, Inc., Maryland, USA.

### Animals

Male 7–8 weeks old Sprague Dawley (SD) rats (180–200 g) were supplied by Harlan Laboratories Inc., Bicester, Oxfordshire, UK. Rats were fed on a chow diet supplied by SDS Ltd, Witham, Essex, UK. Rats were kept in communal cages at temperatures of 19–22 °C, relative air humidity of 45–65% and a light : dark cycle of 12 : 12 hours (lights on at 7 am) with unrestricted access to diet and water. Rats were acclimatised to these conditions for at least 5 days prior to the start of the study. Animals were weighed daily and monitored for signs of ill health. Throughout the study we closely monitored the rats for any signs of distress using the ‘distress scoring sheet’. This included observing the appearance, natural behaviour, hydration status, clinical signs, provoked behaviour, and response to touch. Animals were observed and scored on these parameters. The animal house team also monitored the rats for any signs of distress during the fasting period. Moreover, many of the metabolomics papers conducted similar fasting methods, from 6 to 24 hours, after treating their animals with drugs.^25,43,44^ All animal procedures were performed in accordance with the Guidelines for Care and Use of Laboratory Animals of the Home Office Act ‘Code of Practice for the Housing Care of Animals used in Scientific Procedures’, UK Home Office (1986). University and experiments were approved by the animal ethics committee of Bloomsbury Ethics Committee.

### Sample collection and preparation

Rats (*n* = 15) were dosed by intraperitoneal injection (I.P.) with either 0.9% saline (vehicle untreated; *n* = 5) or tetracycline dissolved in 0.9% saline at a dose level of either 62.5 mg kg^−1^ body weight (*n* = 5) or 125 mg kg^−1^ body weight (*n* = 5). The concentration of tetracycline was adjusted so that the maximum volume received by an animal was 0.3 mL. Control animals received 0.3 mL saline solution only. Tetracycline doses were prepared fresh and used within 30 minutes of preparation. The rats were injected once a day for two days. After the second dose animals were placed in metabolism cages and urine collected over 12 hours. Urine was collected over ice and stored at −80 °C until later analysis. In the metabolism cages rats could access water but not diet. Upon removal from the metabolism cages, rats were killed by I.P. injection of pentobarbital sodium, and exsanguinated from the abdominal aorta. The livers were harvested and weighed. All the liver lobes were removed and placed in either formalin or frozen in liquid nitrogen and stored at −80 °C until later analysis.

### Histopathologic examination

The right and left liver lobules were excised and fixed in formalin. These liver lobules were processed, stained with H&E or ORO. H&E was processed and used for morphological analysis conducted by the pathologist at the Royal Veterinary College (RVC). ORO was used for the assessment of lipids in the hepatic cells carried out by Histologix Ltd. Epifluorescent microscope, EvosFL Auto was used to take photomicrographs of both the H&E and ORO liver tissues.

### Sample preparation for the triglyceride assay

Frozen liver samples were ground to a powder under liquid nitrogen, and 150–250 mg transferred to a microfuge tube. 400 μL ethanolic KOH was added to the powdered liver, samples were vortexed and incubated at 55 °C. After 2 hours 300 μL of ethanolic KOH was added, vortexed and placed into the incubator at 55 °C overnight. Samples were centrifuged at 13 000 rpm for 5 minutes and the supernatant removed. Water : ethanol (1 : 1) solution was added to adjust the volume to 1000 μL. 200 μL of the sample was transferred to a new Eppendorf tube and 215 μL of 1 M MgCl_2_ was added. The samples were vortexed, left on ice for 10 minutes and centrifuged at 13 000 rpm for 5 minutes.

### Triglyceride assay

Analysis of liver triglyceride content was performed by measuring the glycerol released from the saponification in ethanolic KOH using glycerol oxidase (Sigma kit F6428).^45^ A calibration curve was constructed using glycerol standards (Sigma G7793). Glycerol content was measured spectrophotometrically by the change in absorbance of oxidized 4-aminophenazone at 540 nm and expressed as triglyceride equivalents.

### ^1^H NMR spectroscopic sample preparation

The urine samples were thawed and centrifuged at 13 000 rpm for 5 minutes. 200 μL of buffer solution (0.2 M Na_2_HPO_4_/0.2 M NaH_2_PO_4_) was added to 500 μL of urine before centrifugation at 13 000 rpm for 10 minutes. Urine sample mixtures and 50 μL of a standard solution of 1 mM TSP in D_2_O solution, was transferred to a 5 mm NMR tube. The TSP provided a chemical shift reference (*δ* = 0.0) and D_2_O provided a deuterium lock signal for the NMR spectrometer.

### ^1^H NMR spectra experiments

Urine samples were analysed by one-dimensional (1D) and two-dimensional (2D) ^1^H NMR spectroscopy. ^1^H NMR spectra were acquired using a 500 MHz Bruker DRX-500 spectrometer equipped with QNP cryoprobe. Using 1D profiling method which employed presaturation and 90° pulse calibration using pulsecal on each sample. Experimental methods were performed using modified procedure as described by Guo *et al.* (2015) and Tang *et al.* (2017). For more detail methods regarding ^1^H NMR spectroscopy, see ESI.†

### ^1^H NMR and 2D ^1^H–^1^H TOCSY data analysis with metabolite identification

All ^1^H NMR spectra were manually corrected for baseline and phase using TopSpin version 4.0.4 (Bruker Analytik, Rheinstetten, Germany). Identification of metabolites based on the chemical shifts that changed between treated and control samples, was conducted by visual comparison of the ^1^H NMR data collected against spectra obtained in previously published literature and databases such as KEGG pathway database (http://www.genome.jp/kegg/) and Human Metabolome Database (HMDB) (http://www.hmdb.ca/). The correlations observed in the 2D ^1^H–^1^H TOCSY spectra were used to confirm the assignments obtained from 1D ^1^H NMR spectra.

### Metabolite extraction experiments for ESI-LC-MS

Urine samples were diluted 1 : 1 using a mixture composed of acetonitrile/methanol/water 40 : 40 : 20 v/v/v and vortexed. This solution was further diluted by 1 : 1 with acetonitrile containing 0.2% acetic acid, vortexed and spun 10 minutes at 17 000 × *g* at 4 °C to pellet insoluble materials. Four μL of the resulting supernatants were then injected into the LC-MS.

### ESI-LC-MS experiments

Aqueous normal phase liquid chromatography was performed using an Agilent 1290 Infinity II LC system equipped with a binary pump, temperature-controlled auto-sampler (set at 4 °C) and temperature-controlled column compartment (set at 25 °C) containing a Cogent Diamond Hydride Type C silica column (150 mm × 2.1 mm; dead volume 315 μL). A flow rate of 0.4 mL min^−1^ was used. Elution of polar metabolites was carried out using solvent A consisting of deionized water (resistivity ∼ 18 MW cm) and 0.2% acetic acid, and solvent B consisting of 0.2% acetic acid in acetonitrile. The following gradient was used: 0 min 85% B; 0–2 min 85% B; 3–5 min to 80% B; 6–7 min 75% B; 8–9 min 70% B; 10–11 min 50% B; 11.1–14 min 20% B; 14.1–25 min hold 20% B followed by a 5 min re-equilibration period at 85% B at a flow rate of 0.4 mL min^−1^. Accurate mass spectrometry was carried out using an Agilent Accurate Mass 6545 QTOF apparatus. Experimental methods were performed using modified procedure as described by Lobato-Marquez *et al.* (2019), Reboll-Ramirez *et al.* (2019) and Liu *et al.* (2020). For more detail methods regarding ESI-LC-MS, see ESI.†

### ESI-LC-MS data analysis and metabolite identification

Metabolites were identified by comparing the metabolite's feature exact mass (*m*/*z*) obtained from LC-MS against putative mass values in the METLIN database (http://metalin.scrpps.edu), within a mass difference of <10 ppm. The MS/MS fragmentation analysis of the metabolite, was compared to plausible biomarkers suggested by competitive fragmentation modelling for metabolite identification (CFM-ID) and Molecular Structure Correlator using the METLIN database (Agilent). Biochemical pathways involved were found using KEGG pathway database (http://www.genome.jp/kegg/) and Human Metabolome Database (HMDB) (http://www.hmdb.ca/).

### Statistical analysis

Metabolites obtained from both ^1^H-NMR and ESI-LC-MS/MS data are further validated by Kruskal–Wallis test using statistical software STATA 15. *P*-Values < 0.05 are selected as statistically significant.

## Results

**Fig. 1:**
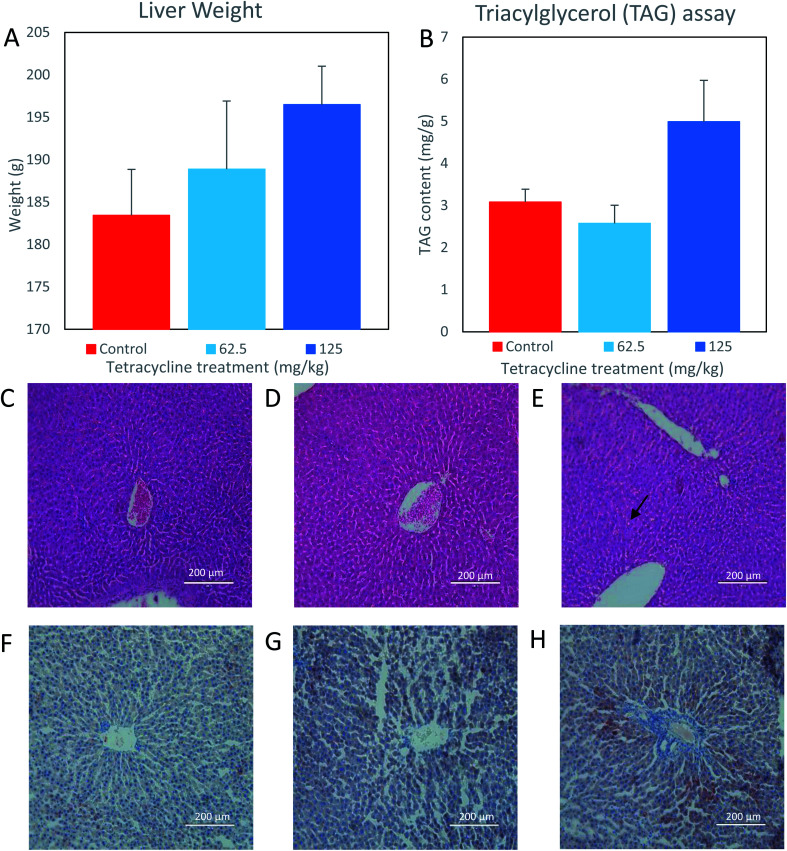
Liver structure and fat content of rats treated with tetracycline. (A) Mean liver weight increased in both the 62.5 mg kg^−1^ and 125 mg kg^−1^ tetracycline treated rats. (B) Mean triacylglycerol content increased in the 125 mg kg^−1^ tetracycline treated rats' liver (*n* = 5) and a decrease in the 62.5 mg kg^−1^ tetracycline rats' liver (*n* = 5). Data are mean ± SEM (Kruskal–Wallis). (C–E) Hematoxylin and eosin stain and (F–H) oil red O stain. Histological images (200 μm) of rats exposed to (C and F) control (D and G) 62.5 mg kg^−1^ tetracycline treated and (E and H) 125 mg kg^−1^ tetracycline treated. Refer to method section for Experimental details and these panels are representative of the 5 rats in each group.

For liver weight analysis, the observed means (SEM) of control, 62.5 mg kg^−1^, and 125 mg kg^−1^ tetracycline-treated rat samples were 183.4 g (5.4), 188.9 g (8.0), and 196.5 g (4.5), respectively. Respective observed medians (range) are 180.5 g (170.4–203.3), 184.3 g (173.9–219.4) and 198.3 g (185.3–210.8). While there is no statistically significant difference between any of the three groups (*p* = 0.16), a visual inspection of the data (Fig. 1A) suggests a higher average value of liver weight in the 125 mg kg^−1^ tetracycline-treated rats compared with control group (mean diff.: 13.06 (95% CI: −5.93 to 32.05)) and 62.5 mg kg^−1^ (mean diff.: 5.44 (95% CI: −13.55 to 24.43)).

For the analysis of triacylglycerol content per gram of liver, the observed means (SEM) of control, 62.5 mg kg^−1^, and 125 mg kg^−1^ tetracycline-treated rat samples, are 3.08 mg g^−1^ (0.30), 2.57 mg g^−1^ (0.43), and 4.99 mg g^−1^ (0.98), respectively. Respective observed medians (range) are 2.97 mg g^−1^ (2.29–4.04), 2.54 mg g^−1^ (1.51–4.07) and 5.76 mg g^−1^ (2.73–7.79). Again, there is no statistically significant difference between any of the three groups (*p* = 0.14). However, visual inspection of the data (Fig. 1B) suggests a higher average value of liver triglyceride compared with control group (mean diff.: 1.91 (95% CI: = –0.08 to 3.90)) and 62.5 mg kg^−1^ (mean diff.: −0.51 (95% CI: −2.50 to 1.48)). Histological examination of control and 62.5 mg kg^−1^ tetracycline-treated rat liver tissues exhibited normal lobular architecture (Fig. 1C and D). However, the liver morphology from 125 mg kg^−1^ tetracycline-treated rats (Fig. 1E) displayed mild hepatocellular vacuolation indicated by arrows, potentially containing either glycogen or fat. This is further supported by ORO staining as there was an increase uptake of stain suggesting an increase of intracytoplasmic triacylglycerol content in the 125 mg kg^−1^ tetracycline-treated rats (Fig. 1H), in comparison to control and 62.5 mg kg^−1^ tetracycline-treated rats (Fig. 1F and G). The combined analysis of histological ORO staining and direct measurement of triacylglycerol content, suggests that tetracycline exposure at 125 mg kg^−1^ dosage induces steatosis.

### ^1^H NMR based metabolomics analysis and metabolite identification

**Fig. 2:**
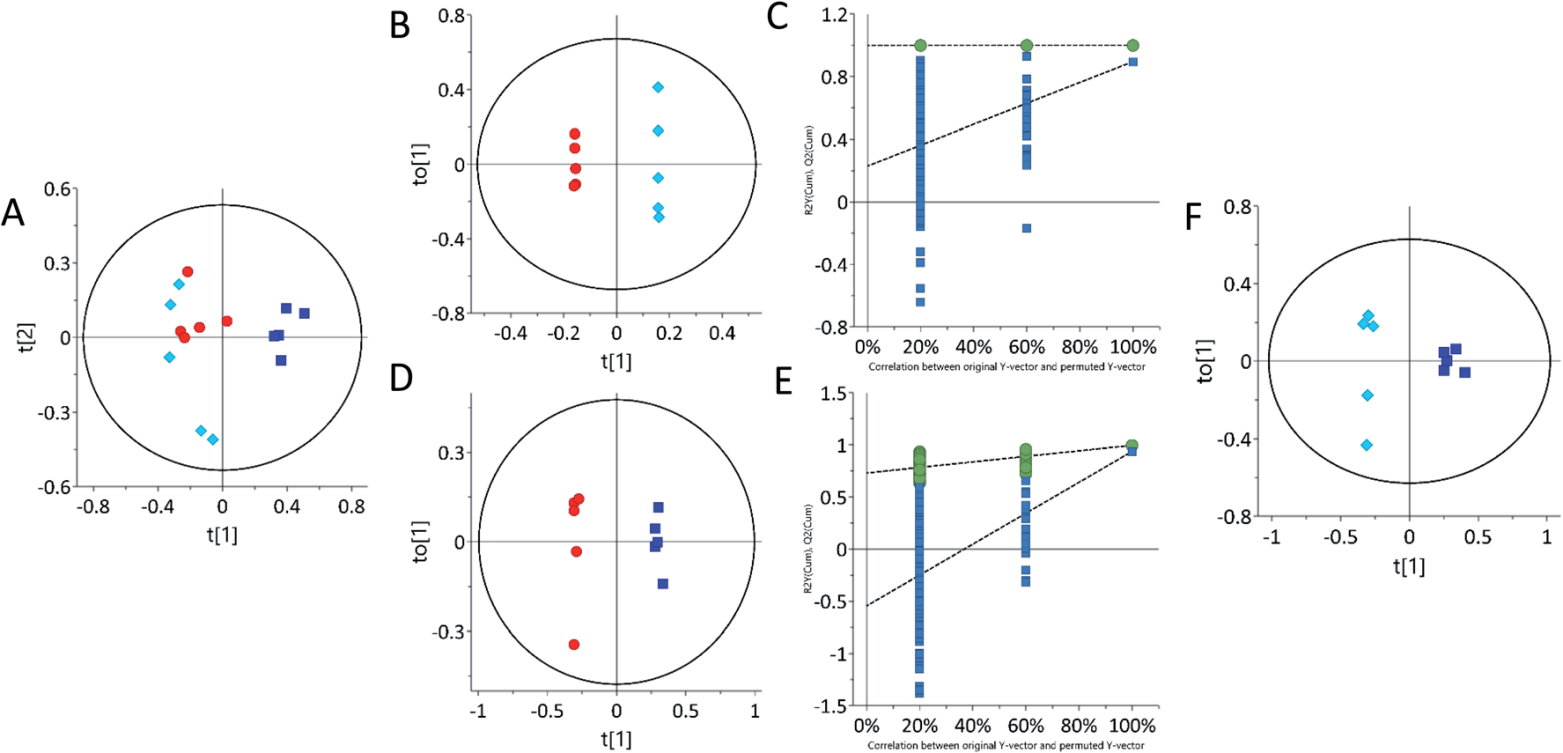
Principal component analysis (PCA) and orthogonal partial least-squares discriminant analysis (OPLS-DA) of urine samples acquired by ^1^H NMR spectra showing separation of control and treated. (A) PCA scores plot of urine, (B, D and F) OPLS-DA scores plot of urine. (C and E) Permutation test plots (200 permutations) for control *vs.* 62.5 mg kg^−1^ (*R*^2^ 0.99, 0.0; *Q*^2^ 0.0, 0.87) and control *vs.* 125 mg kg^−1^ (*R*^2^ 0.0, 0.99; *Q*^2^ 0.0, 0.91). Each point on the scores plot represents one sample. Control samples are represented by red circles (*n* = 5), 62.5 mg kg^−1^ samples (*n* = 5) are represented by light blue diamonds (*n* = 5) and 125 mg kg^−1^ are represented by dark blue squares (*n* = 5). See Table S1 ESI† for *R*^2^*X* (cum), *R*^2^*Y* and *Q*^2^ (cum) details.

Multivariate statistical analysis by principal component analysis (PCA) scores plot from ^1^H-NMR spectra revealed differences between the control and 62.5 mg kg^−1^ or 125 mg kg^−1^ tetracycline-treated rats. The urine PCA scores plot showed three different groups, indicating that tetracycline induced changes in the rats' metabolic profile (Fig. 2A). An orthogonal partial least squares discriminant analysis (OPLS-DA) scores plot from ^1^H-NMR further suggests metabolite differences between control and treated, and additionally differentiating between the two administered concentrations of tetracycline. This is shown on OPLS-DA scores plot urinary data (Fig. 2B and D). The urine OPLS-DA score plots consistently showcased tetracycline-treated groups as clustering away from controls. In addition, urine OPLS-DA scores plot showed that the 125 mg kg^−1^ tetracycline-treated group clustered away from the 62.5 mg kg^−1^ tetracycline-treated group (Fig. 2F). The parameters (*R*^2^*X*, *Q*^2^) for each model indicated satisfactory goodness of prediction and goodness of fit (Fig. S1 ESI† and Table 1). The pattern differences correlate with steatosis severity as judged by Fig. 1 data and highlights that there is a clear distinction of a pattern difference at the lower dose level (62.5 mg kg^−1^) when histological examination and TAG analysis were unable to show any differences. *S*-Plot and variable importance in the projection (VIP) value plot (Table S1 ESI†) were then examined with VIP scores larger than 1.0 selected. The VIP plot ranks the important variables from the OPLS-DA scores plot.^46^ Chemical shifts and peak multiplicities were assigned by analysing the urine ^1^H NMR spectra. Metabolites were then identified using information of peaks from human metabolome database (HMDB) and previously published publications, then further confirmed by correlations found in 2D ^1^H–^1^H TOCSY NMR spectra. The presence of three urinary metabolites varied significantly (*p* < 0.05) between control and treated groups (Table 1). There were however many other statistically significant metabolites that remained unidentified partly due to peak overlap and low concentrations (Table S2 ESI†). Some of the chemical shifts of the identified metabolites were confirmed using 2D ^1^H–^1^H TOSCY NMR spectroscopy (Fig. S2 ESI†).

**Table 1:** Filtered list of metabolites detected by 1D ^1^H NMR spectra. Metabolites detected by *S*-plot and VIP plot analysis (see Fig. S1 ESI) of 1D ^1^H NMR spectra of urine from rats treated by either 62.5 mg kg^−1^ or 125 mg kg^−1^ tetracycline. Refer to method section for Experimental details. Values differing significantly according to *S*-plot and VIP plot are shown. Doublet (d), triplet (t) and multiplet (m)

Tetracycline treated samples							
Metabolites	Chemical shift (ppm)	Multiplicities	*P*-Value	62.5 mg kg^−1^		125 mg kg^−1^	
				Fold change	VIP	Fold change	VIP
Hippurate	7.84	d	0.016	0.904	—	3.209	2.275
Citrate	2.54	d	0.011	0.926	—	1.480	2.279
Lysine	1.98	m	0.016	0.924	1.377	0.868	1.206

Two statistically significant urinary metabolites that were detected by ^1^H NMR increased after tetracycline treatment of rats with 125 mg kg^−1^: hippurate ∼ 220.9 and citrate ∼ 48.0%. In addition, lysine decreased by ∼7.6% and ∼13.2% after tetracycline treatment of rats with 62.5 mg kg^−1^ and 125 mg kg^−1^, respectively. Relationships between altered metabolic profiles and associated biological pathways were identified using the KEGG pathway database and HMDB (Table 3). The identified altered pathways include changes in TCA cycle, phenylalanine and the amino acid metabolism pathway.

### LC-MS/MS based metabolomics analysis and metabolite identification

**Fig. 3:**
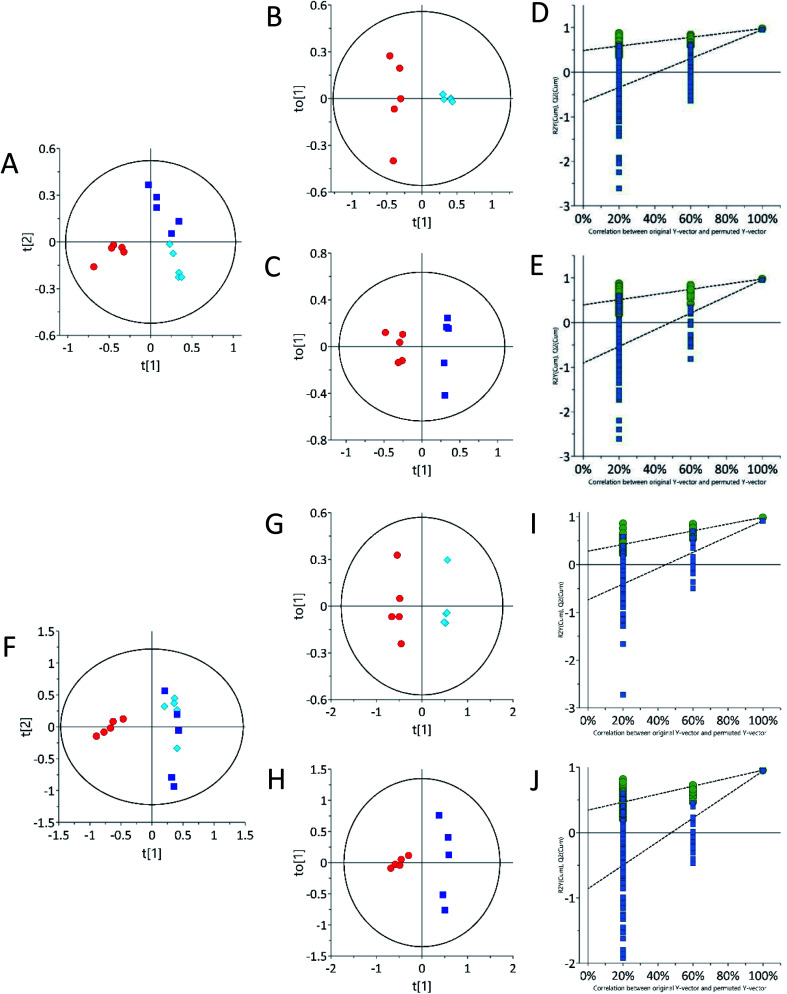
Principal component analysis (PCA) urine samples acquired by ESI-LC-MS showing clustering based on differential treatments. (A) ESI+ and (F) ESI− PCA scores plot of urine, (B and C) ESI+ and (G and H) ESI− OPLS-DA scores plot of urine, (D and E) ESI+ and (I and J) ESI− permutation test plots (200 permutations) for control *vs.* 62.5 mg kg^−1^ (*R*^2^ 0.0, 0.99; *Q*^2^ 0.0, 0.97), control *vs.* 125 mg kg^−1^ (*R*^2^ 0.0, 0.91; *Q*^2^ 0.0, 0.86). Control samples (*n* = 5) are represented by red circles, 62.5 mg kg^−1^ (*n* = 5) samples are represented by light blue diamonds and 125 mg kg^−1^ (*n* = 5) are represented by dark blue squares.

Urine sample metabolic profiles were also obtained using LC-ESI-LC-MS in both positive and negative ion scan modes. According to the ESI-LC-MS PCA scores plot (Fig. 3A and F), both 62.5 mg kg^−1^ and 125 mg kg^−1^ tetracycline-treated rats grouped away from control in both the positive and negative ion modes. Urine samples in ESI− and ESI+, the 62.5 mg kg^−1^ and 125 mg kg^−1^ tetracycline-treated groups (Fig. 3B, C, G and H) were clustered separately away. Distinct separation of the urinary samples from both tetracycline doses were observed. This supports the pattern differences seen between tetracycline doses in ^1^H NMR (Fig. 2). This highlights that there is a clear distinction of metabolite pattern difference between controls and the lower tetracycline dose level (62.5 mg kg^−1^) when histological examination and TAG analysis were unable to show any differences. Analysis by ESI-LC-MS/MS showed that the presence of three urinary metabolites varied significantly (*p* < 0.05) between control and treated groups (Table 2). Structures of the detected metabolites were determined by MS/MS fragmentation (Fig. S3 ESI†), accurate mass, together with isotope ratio and isotope abundance.^47^ METLIN, competitive fragmentation modelling for metabolite identification (CFM-ID), and MassHunter databases, were then used to confirm plausible biomarkers. Urinary metabolite detected by ESI-LC-MS/MS to have increased after tetracycline treatment of 62.5 mg kg^−1^ and 125 mg kg^−1^ was proline ∼ 368.4.2% and ∼196.6%, respectively. Two urine metabolites instead decreased after tetracycline treatment of 62.5 mg kg^−1^ and 125 mg kg^−1^: taurine ∼ 91.5% and ∼86.3% and inosine ∼83.9% and ∼66.2%, respectively. Adenosine, guanine and xanthurenic acid were also detected *via* ESI-LC-MS/MS (Fig. S3 ESI†). Xanthurenic acid was found to be increased after tetracycline treatment of 62.5 mg kg^−1^ and 125 mg kg^−1^ by ∼13.0% and ∼23.5%, respectively. Although, this metabolite was not statistically significant it should be noted that xanthurenic acid concentration correlated with liver damage as tetracycline dose concentration increased. The concentrations of metabolites, adenosine and guanine, were unable to be calculated. Relationships between altered metabolic profiles and associated biological pathways were identified using the KEGG pathway database and HMDB (Table 3). The identified altered pathways include changes in purine, amino acid and gut metabolism pathway.

**Table 2:** Filtered list of metabolites detected by ESI-LC-MS/MS from urine of rats. Rats treated with either 62.5 mg kg^−1^ (*n* = 5) or 125 mg kg^−1^ (*n* = 5) tetracycline. Refer to methods for Experimental details

*m*/*z*	Mass	Identified metabolite	Formula	Theoretical mass	*Δ*_ppm_	RT (min)	*P*-Value	62.5 mg kg^−1^		125 mg kg^−1^	
								Fold change	VIP	Fold change	VIP
116.071	115.06	Proline	C_5_H_9_NO_2_	115.063	4	1.808	0.026	4.684	3.66	2.966	2.95
269.088	268.08	Inosine	C_10_H_12_N_4_O_5_	268.081	1	2.237	0.005	0.161	0.97	0.338	0.28
126.021	125.01	Taurine	C_2_H_7_NO_3_S	125.014	2	3.279	0.002	0.085	1.99	0.137	2.11

**Table 3:** Summary of potential metabolites and their associated metabolic pathways detected by ^1^H NMR and LC-ESI-MS/MS of urine from tetracycline treated rats

Pathways	Metabolite	Detection
TCA cycle	Citrate	NMR
Phenylalanine metabolism	Hippurate	NMR
Gut metabolism	Xanthurenic acid	LC-MS/MS
Lysine degradation	Lysine	NMR
Taurine and hypotaurine metabolism	Taurine	NMR
Purine metabolism	Inosine	LC-MS/MS
Arginine and proline metabolism		
Arginine and proline metabolism	Proline	LC-MS/MS

## Discussion

Our study observes that metabolic profiling can distinguish between impending steatosis and liver damage better than histopathology and biochemical measurement of triglyceride which both require a biopsy. Urine metabolomics was conducted using a combination of analytical techniques to evaluate changes in metabolite levels. Metabolites were analysed in urine from tetracycline treated rats that have shown *via* both histopathology and triglyceride analysis to produce different severity of liver steatotic pathogenesis.^38,48–50^ Using both ^1^H-NMR spectroscopy and ESI-LC-MS/MS spectrometry, showcased distinct metabolomic differences between control and 62.5 mg kg^−1^ and 125 mg kg^−1^ tetracycline-treated rats. Whilst spectral data analysis by these two techniques identified metabolites, with some differences that are unavoidable due to the different levels of detection and principles of identification applied by ^1^H-NMR and ESI-LC-MS/MS. Therefore, integration of results obtained by both platforms maximised the ability to detect metabolites with different concentrations in control and treated samples.

Previous studies demonstrated the steatogenic effect of tetracycline in decreasing mitochondrial β-oxidation of fatty acids,^21,24–27,31,51^ altering the expression of genes related to lipid metabolism, which downstream elevates cholesterol and triglyceride biosynthetic activity.^24,27,52,53^ In this current study, there is no evidence from this data for an increase in TAG synthesis. However, it can be proposed that the redirection of fatty acids to TAG synthesis is the result of these inhibitory actions as part of the mechanism of tetracycline induced steatosis shown by higher liver TAG content in the hepatic cells of the 125 mg kg^−1^ tetracycline treated rats *via* oil red staining.

Through ^1^H NMR spectroscopy, results from our study highlight increased hippurate and citrate levels in the 125 mg kg^−1^ tetracycline treated rats. Hippurate, is the product of benzoate conjugation with glycine in the liver ready for excretion in urine and is an ATP-dependent reaction^54–56^ and associated with gut metabolism.^57,58^ Hippurate has been reported as an indicator of hepatic damage or alteration to liver function as urinary hippurate was observed to be decreased after animals received three different steatogenic compounds.^19,56,59^ Hippurate could potentially be a biomarker, however, further investigation is required.

Another mechanism whereby steatosis occurs involves carnitine, an amino acid derived from the substrate lysine, used as a transport shuttle for free fatty acids into the mitochondria. Tetracycline is known to reduce expression levels of carnitine acyl transferase 1 (CAT1).^19^ CAT1 is the enzyme responsible for the acylation of carnitine in mitochondrial fatty acid transport, and reduced expression of CAT1 consequently leads to a reduction in β-oxidation.^19^ Furthermore, studies examining the effects of lysine-deficient diets induced by different drugs, report the reduction of lysine as a contributing factor in the development of steatosis.^60^ Our tetracycline treated rats featured a decrease in lysine and an increase in proline. We conclude that in this study although tetracycline affects the lysine and proline pathways, further investigation is required to understand the connection with steatosis.

The amino acid taurine attenuates the development of steatosis, by inhibiting oxidative stress through action as an antioxidant.^49,61^*In vitro* and *in vivo* studies from Murakami *et al.* (2018) indirectly support this, reporting supplementation with taurine as lessening lipid accumulation by inhibiting oxidative stress. A decrease of taurine concentrations was also observed in another steatogenic drug, sodium valproate, which could be an important metabolite to investigate in future studies of steatogenic induced liver disease.^20^ Similarly, data from our ^1^H NMR and ESI-LC-MS/MS illustrates an overall decrease in taurine suggesting either a decrease in the production of taurine^62^ or being spared from excretion as it is used as an antioxidant in response to tetracycline, which both potentially contribute to development of steatosis. In this current study, it can be proposed that reduced taurine levels therefore are reflective of a damaged liver or used as an antioxidant where lipids cause oxidative stress by undergoing peroxidation which then leads to the production of reactive oxygen species (ROS).^54^ Asha *et al.* (2007) details the instability of ROS and their propensity to react with lipids, proteins, DNA and other cellular macromolecules. Data from our ESI-LC-MS/MS reveals an intermediate from the purine metabolic pathway in our tetracycline treated rats, showing a decrease in urinary inosine. Adenosine and guanine were also identified by ESI-LC-MS/MS. Therefore, these results are suggestive of reactions between ROS and DNA, causing the alterations in purine metabolism, although, further investigation is required to understand the connection with steatosis.

This study has both strengths and limitations. We acknowledge that our sample size is small, although adequate for proving our objectives. Despite being underpowered, our pilot study in fact supports changes in biochemical pathways which, according to multiple previous studies, are understood to result from tetracycline-induced steatosis. This study, therefore, serves as a platform for further studies to validate not only the potential biomarkers presented here but also the novel findings we propose.

## Conclusion

In the livers of rats treated with 125 mg kg^−1^ tetracycline H&E stained photomicrographs revealed changes in the lobular architecture, whilst rats treated with a lower dose (62.5 mg kg^−1^) displayed architecture similar those of control animals. Interestingly, data from our ^1^H NMR and ESI-LC-MS/MS was able to distinguishing between controls, low dose and high dose treated animals. H&E and ORO stained photomicrographs and even biochemical measurements of liver triacylglycerol content instead failed to produce similar outcomes. Results therefore show that coupling the highly quantitative and reproducible nature of ^1^H NMR with the high specificity and sensitivity of ESI-LC-MS/MS based metabolomics, to perform urine analysis, ultimately offers a more comprehensive understanding of the underlying physiological pathways known to be altered by tetracycline. Our study, in fact, demonstrates that metabolomics can detect changes in the urine metabolome prior to observable changes in liver morphology.

Collectively, integration of ^1^H NMR and ESI-LC-MS/MS based metabolomics identified a number of putative biomarkers and metabolic pathways that appear to be associated with steatotic development induced by tetracycline. Data analysis using ^1^H NMR revealed complementary and distinct metabolites corroborated by ESI-LC-MS/MS. We identified lysine as possible liver specific biomarkers, since the concentration correlated with the increase in dosing from 62.5 mg kg^−1^ to 125 mg kg^−1^, which indicates correlation with the extent of liver damage. Although exact alterations to the metabolic pathways observed were difficult to identify, the data highlighted how impairments in several metabolic pathways may ultimately contribute to the complexity of the disease. Whilst inosine was observed to change, further investigation is required to understand the connection with steatosis.

Given these results, the combination of ^1^H NMR and ESI-LC-MS/MS provided a suitable platform for studying the pathogenesis of steatosis in conditions where tissue staining techniques are inadequate. Although results from this investigation are produced from a small sample size, the findings we present build upon previously discovered knowledge and are therefore supported by various preceding studies. Results from this investigation can hence be used as a pilot study laying the foundation for future investigation of other steatogenic-specific biomarkers, for early clinical disease prediction or diagnosis. This information holds much promise towards the use of metabolomics as a less-invasive tool for earlier detection of steatosis, compared to detection by liver biopsy.

## Conflicts of interest

The authors declare that there are no conflicts of interest.

## Supplementary Material

RA-010-D0RA06577C-s001

## References

[cit1] Loomba R., Sanyal A. J. (2013). The global NAFLD epidemic. Nat. Rev. Gastroenterol. Hepatol..

[cit2] Nassir F. (2015). *et al.*, Pathogenesis and Prevention of Hepatic Steatosis. Gastroenterol. Hepatol..

[cit3] Rabinowich L., Shibolet O. (2015). Drug Induced Steatohepatitis: An Uncommon Culprit of a Common Disease. BioMed Res. Int..

[cit4] AlShaalan R. (2015). *et al.*, Nonalcoholic fatty liver disease: noninvasive methods of diagnosing hepatic steatosis. Saudi J. Gastroenterol..

[cit5] Neuschwander-Tetri B. A. (2005). Nonalcoholic steatohepatitis and the metabolic syndrome. Am. J. Med. Sci..

[cit6] Amacher D. E., Chalasani N. (2014). Drug-induced hepatic steatosis. Semin. Liver Dis..

[cit7] Ramachandran R., Kakar S. (2009). Histological patterns in drug-induced liver disease. J. Clin. Pathol..

[cit8] Yang X. , *et al.*, Hepatic toxicity biomarkers, in Biomarkers in Toxicology, 2014, pp. 241–259

[cit9] Michalopoulos G. K. (2007). Liver regeneration. J. Cell. Physiol..

[cit10] Loomba R., Sanyal A. J. (2013). The global NAFLD epidemic. Nat. Rev. Gastroenterol. Hepatol..

[cit11] Botros M., Sikaris K. A. (2013). The de ritis ratio- the test of time. Clin. Biochem. Rev..

[cit12] Poynard T. (2005). *et al.*, The diagnostic value of biomarkers (SteatoTest) for the prediction of liver steatosis. Comp. Hepatol..

[cit13] Mehta S. R. (2008). *et al.*, Non-invasive means of measuring hepatic fat content. World J. Gastroenterol..

[cit14] Gerstenmaier J. F., Gibson R. N. (2014). Ultrasound in chronic liver disease. Insights Imaging.

[cit15] Stefan N., Häring H.-U., Cusi K. (2019). Non-alcoholic fatty liver disease: causes, diagnosis, cardiometabolic consequences, and treatment strategies. Lancet Diabetes Endocrinol..

[cit16] Chang H. (2017). *et al.*, Identification of key metabolic changes during liver fibrosis progression in rats using a urine and serum metabolomics approach. Sci. Rep..

[cit17] Browning J. D. (2004). *et al.*, Prevalence of hepatic steatosis in an urban population in the United States: impact of ethnicity. Hepatology.

[cit18] Grieco A. (2005). *et al.*, Fatty liver and drugs. Eur. Rev. Med. Pharmacol. Sci..

[cit19] Tagliatti V., Colet J. M. (2016). Drug-Induced Impairment of Mitochondrial Fatty Acid Beta-Oxidation: A Metabonomic Evaluation in Rats. J. Med. Genomics.

[cit20] Amacher D. E. (2011). Strategies for the early detection of drug-induced hepatic steatosis in preclinical drug safety evaluation studies. Toxicology.

[cit21] Begriche K. (2011). *et al.*, Drug-induced toxicity on mitochondria and lipid metabolism: mechanistic diversity and deleterious consequences for the liver. J. Hepatol..

[cit22] Cuykx M. (2018). *et al.*, Metabolomics profiling of steatosis progression in HepaRG((R)) cells using sodium valproate. Toxicol. Lett..

[cit23] Chopra I., Roberts M. (2001). Tetracycline antibiotics: mode of action, applications, molecular biology, and epidemiology of bacterial resistance. Microbiol. Mol. Biol. Rev..

[cit24] Choi Y. J. (2015). *et al.*, Increased hepatic fatty acid uptake and esterification contribute to tetracycline-induced steatosis in mice. Toxicol. Sci..

[cit25] Letteron P. (2003). *et al.*, Inhibition of microsomal triglyceride transfer protein: another mechanism for drug-induced steatosis in mice. Hepatology.

[cit26] Zimmerman H. J. (2000). Drug-induced liver disease. Clin. Liver Dis..

[cit27] Fréneaux E., Labbe G., Letteron P., Dinh T. L., Degott C., Genève J., Larrey D., Pessayre D. (1988). Inhibition of the mitochondrial oxidation of fatty acids by tetracycline in mice and in man: possible role in microvesicular steatosis induced by this antibiotic. Hepatology.

[cit28] Schultz J. C., Adamson Jr J. S., Workman W. W., Norman T. D. (1963). Fatal liver disease after intravenous administration of tetracycline in high dosage. N. Engl. J. Med..

[cit29] Westphal J. F., Vetter D., Brogard J. M. (1994). Hepatic side-effects of antibiotics. J. Antimicrob. Chemother..

[cit30] Wang G., Li Z., Li H., Li L., Li J., Yu C. (2016). Metabolic Profile Changes of CCl(4)-Liver Fibrosis and Inhibitory Effects of Jiaqi Ganxian Granule. Molecules.

[cit31] Breen K., Schenker S., Heimberg M. (1972). The effect of tetracycline on the hepatic secretion of triglyceride. Biochim. Biophys. Acta.

[cit32] Labbe G., Fromenty B., Freneaux E., Morzelle V., Letteron P., Berson A., Pessayre D. (1991). Effects of various tetracycline derivatives on in vitro and in vivo beta-oxidation of fatty acids, egress of triglycerides from the liver, accumulation of hepatic triglycerides, and mortality in mice. Biochem. Pharmacol..

[cit33] Cano A. (2017). *et al.*, A Metabolomics Signature Linked to Liver Fibrosis in the Serum of Transplanted Hepatitis C Patients. Sci. Rep..

[cit34] Deboyser D. (1989). *et al.*, Investigation into the Mechanism of Tetracycline-Induced Steatosis - Study in Isolated Hepatocytes. Toxicol. Appl. Pharmacol..

[cit35] Gitto S., Schepis F., Andreone P., Villa E. (2018). Study of the Serum Metabolomic Profile in Nonalcoholic Fatty Liver Disease: Research and Clinical Perspectives. Metabolites.

[cit36] van Ginneken V. (2007). *et al.*, Metabolomics (liver and blood profiling) in a mouse model in response to fasting: a study of hepatic steatosis. Biochim. Biophys. Acta.

[cit37] Bril F. , Kalavalapalli S., Duffin K. L., Hartman M. L., Chen Y., Yang Q., Haas J. V., Milligan P. L., Roth K. D. and Cusi K., Use of Plasma Metabolomics and Lipidomics for the Diagnosis of Nonalcoholic Fatty Liver Disease in Type 2 Diabetes, 2018

[cit38] Li H. (2011). *et al.*, A proton nuclear magnetic resonance metabonomics approach for biomarker discovery in nonalcoholic fatty liver disease. J. Proteome Res..

[cit39] Serkova N. J. (2006). *et al.*, Metabolic profiling of livers and blood from obese Zucker rats. J. Hepatol..

[cit40] Song X. (2013). *et al.*, (1)H NMR-based metabolomics approach to evaluate the effect of Xue-Fu-Zhu-Yu decoction on hyperlipidemia rats induced by high-fat diet. J. Pharm. Biomed. Anal..

[cit41] Zhao L. (2011). *et al.*, 1H NMR-Based Metabonomic Analysis of Metabolic Changes of Serum and Liver in Zucker Obese Rats. Anal. Lett..

[cit42] Iruzubieta P. (2015). *et al.*, The Need for Biomarkers in Diagnosis and Prognosis of Drug-Induced Liver Disease: Does Metabolomics Have Any Role?. BioMed Res. Int..

[cit43] Ichimura M. (2017). *et al.*, A diet-induced Sprague-Dawley rat model of nonalcoholic steatohepatitis-related cirrhosis. J. Nutr. Biochem..

[cit44] An L. (2016). *et al.*, Dynamic metabolic profiling of urine biomarkers in rats with alcohol induced liver damage following treatment with ZhiZiDaHuang decoction. Mol. Med. Rep..

[cit45] Norris A. W. (2003). *et al.*, Muscle-specific PPARγ-deficient mice develop increased adiposity and insulin resistance but respond to thiazolidinediones. J. Clin. Invest..

[cit46] Trivedi D. K., Iles R. K. (2012). The Application of SIMCA P+ in Shotgun Metabolomics Analysis of ZIC®HILIC-MS Spectra of Human Urine - Experience with the Shimadzu IT-T of and Profiling Solutions Data Extraction Software. J. Chromatogr.
Sep. Tech..

[cit47] Sumner L. W. (2007). *et al.*, Proposed Minimum Reporting Standards for Chemical Analysis Chemical Analysis Working Group (CAWG) Metabolomics Standards Initiative (MSI). Metabolomics.

[cit48] Chan T. S. (2018). *et al.*, Upregulation of Krebs cycle and anaerobic glycolysis activity early after onset of liver ischemia. PLoS One.

[cit49] Zhao H. (2017). *et al.*, Pyrazinamide-induced hepatotoxicity and gender differences in rats as revealed by a (1)H NMR based metabolomics approach. Toxicol. Res..

[cit50] Shin O. H., Mar M. H., Albright C. D., Citarella M. T., da Costa K. A., Zeisel S. H. (1997). Methyl-group donors cannot prevent apoptotic death of rat hepatocytes induced by choline-deficiency. J. Cell. Biochem..

[cit51] Du Buy H. G., Showacre J. L. (1961). Selective localization of tetracycline in mitochondria of living cells. Science.

[cit52] Garcia-Canaveras J. C. (2016). *et al.*, A metabolomics cell-based approach for anticipating and investigating drug-induced liver injury. Sci. Rep..

[cit53] Yin H. Q. (2006). *et al.*, Hepatic gene expression profiling and lipid homeostasis in mice exposed to steatogenic drug, tetracycline. Toxicol. Sci..

[cit54] Asha K. K., Sankar T. V., Viswanathan Nair P. G. (2007). Effect of tetracycline on pancreas and liver function of adult male albino rats. J. Pharm. Pharmacol..

[cit55] Gatley S. J. and Sherratt H. S. A., The localization of hippurate synthesis in the matrix of rat liver mitochondria, 1976 10.1042/bst0040525 1001717

[cit56] Kim K.-B. (2008). *et al.*, Metabolomics and biomarker discovery: NMR spectral data of urine and hepatotoxicity by carbon tetrachloride, acetaminophen, and d-galactosamine in rats. Metabolomics.

[cit57] Aron-Wisnewsky J. (2013). *et al.*, Gut microbiota and non-alcoholic fatty liver disease: new insights. Clin. Microbiol. Infect..

[cit58] Dumas M. E., Kinross J., Nicholson J. K. (2014). Metabolic phenotyping and systems biology approaches to understanding metabolic syndrome and fatty liver disease. Gastroenterology.

[cit59] Yavuz A. , Tetta C., Ersoy F. F., D'intini V., Ratanarat R., De Cal M., Bonello M., Bordoni V., Salvatori G., Andrikos E. and Yakupoglu G., Reviews: uremic toxins: a new focus on an old subject, in Seminars in dialysis, 2005, Blackwell Science Inc., Oxford, UK, vol. 183, no. 3, pp. 203–211 10.1111/j.1525-139X.2005.18313.x 15934967

[cit60] Lin H. Y. (2014). *et al.*, Enhanced amelioration of high-fat diet-induced fatty liver by docosahexaenoic acid and lysine supplementations. BioMed Res. Int..

[cit61] Murakami S. (2018). *et al.*, Taurine attenuates the development of hepatic steatosis through the inhibition of oxidative stress in a model of nonalcoholic fatty liver disease in vivo and in vitro. Amino Acids.

[cit62] Wen C. (2019). *et al.*, Taurine is Involved in Energy Metabolism in Muscles, Adipose Tissue, and the Liver. Mol. Nutr. Food Res..

