# Supplementary material for: Early detection of metabolic changes in drug-induced steatosis using metabolomics approaches
Source: RSC Adv. 2020 Nov 11;10(67):41047–57. doi: 10.1039/d0ra06577c (PMC9057704; doi:10.1039/d0ra06577c)
Supplement: RA-010-D0RA06577C-s001 [file RA-010-D0RA06577C-s001.pdf]

Electronic Supplementary Material (ESI) for RSC Advances.
This journal is © The Royal Society of Chemistry 2020

Supplementary Information.

Supporting methods

^{1}H NMR spectroscopy experiment

Urine were analysed by one-dimensional (1D) and two-dimensional (2D) ^{1}H NMR spectroscopy. ^{1}H NMR spectra were acquired using a 500MHz Bruker DRX-500 spectrometer equipped with QNP cryoprobe. A standard pulse sequence was adopted for water suppression with irradiation at the water frequency, with a relaxation delay of 3 seconds and a pulse sequence mixing time of 100 ms. Following four dummy scans, spectra were acquired using 64 scans into 64K points using a spectral width of 10.080 ppm, with an acquisition time of 4.68 seconds, and a total pulse recycle time of 7.68 seconds. The free induction decays (FIDs) were multiplied by an exponential weighting function corresponding to a line broadening of 0.2 Hz prior to Fourier Transform. Lastly, homonuclear total correlation spectroscopy (2D ^{1}H-^{1}H TOCSY) experiments of the untreated urine samples were conducted.

Experimental methods were performed using modified procedure as described by Guo *et al* (2015) and Tang *et al* (2017). All ^{1}H NMR spectra were manually corrected for baseline and phase using TopSpin version 4.0.4 (Bruker Analytik, Rheinstetten, Germany). The TSP resonance at 0 ppm was incorporated as the internal standard for urine. In the urine spectra, the region between δ 6.1 -4.5 and δ 0.2 to -0.2 was set to a zero integral value, removing variability in presaturation water resonance and cross-relaxation effects on the urea and TSP signals. In addition, the tetracycline parent peaks were removed before conducting the multivariate analysis. The data was reduced using AMIX (Bruker Analytik, Rheinstetten, Germany) to regions of 0.04 ppm wide from δ 10.0 to 0.0, producing 250 chemical shift values also known as buckets. The signal intensities within each bucket were integrated in a process known as bucketing or binning. After the data was pre-processed it was imported into Microsoft Excel generating the bucket list. For normalization of the urine volume, integral values of individual metabolites were recalculated as a percentage of the sum of total integrals in a spectrum. Each Excel bucket list was then imported into SIMCA 15 (Simca v. 15, MKS Umetrics AB, Sweden), mean centred and scaled using Pareto scaling for pattern recognition analysis. [43] To reveal metabolite changes in different groups, pattern recognition analysis was performed by conducting principal component analysis (PCA) and orthogonal partial least-squares discriminant analysis (OPLS-DA) using SIMCA 15 (Simca v. 15, Sartorius Umetrics AB, Sweden). Outlier samples that fell outside the Hotelling's T2 plot on the PCA scores plot were individually analysed and excluded from further analysis when necessary. Subsequently, the OPLS-DA were generated to maximise separation between the examined groups and to identify chemical shifts changes between two sample groups. In all models the quality of the model was assessed by validation parameters Q^2 which estimates the predictability of the model and R^2 (cum) which represents goodness of fit. R^2 is defined by R^2X and R^2Y. R^2X describes how the variation in the X matrix (rows/observations) is correlated to the Y variable (e.g. healthy against diseased), and the R^2Y is a measure for degree of separation between Y variables. Loadings S-Plot were used to identify spectral regions responsible for sample separation, while a variable importance in the projection (VIP) plot summarised critical variables within the OPLS-DA scores plot, and their subsequent ranking. VIP values of >1 are most influential for discriminating between 2 sample groups.

LC-ESI-MS experiment

Dynamic mass axis calibration was achieved by continuous infusion, post-chromatography, of a reference mass solution using an isocratic pump connected to an ESI ionization source operated in the positive-ion and negative-ion mode. The nozzle voltage and fragmentor voltage were set at 2,000 V and 100 V, respectively. The nebulizer pressure was set at 50 psig, and the nitrogen drying gas flow rate was set at 5 L/min. The drying gas temperature was maintained at 300°C. The MS acquisition rate was 1.5 spectra/sec, and m/z data ranging from 50 to 1,200 m/z were stored. This instrument enabled accurate mass spectral measurements with an error of less than 5 parts-per-million (ppm), mass resolution ranging between 10,000-25,000 over the m/z range of 121-955, and a 100,000-fold dynamic range with picomolar sensitivity. The data were collected in the centroid 4 GHz (extended dynamic range) mode. Experimental methods were performed using modified procedure as described by Lobato-Marquez *et a*l (2019), Rebollo-Ramirez *et al* (2019) and Liu *et al* (2020).

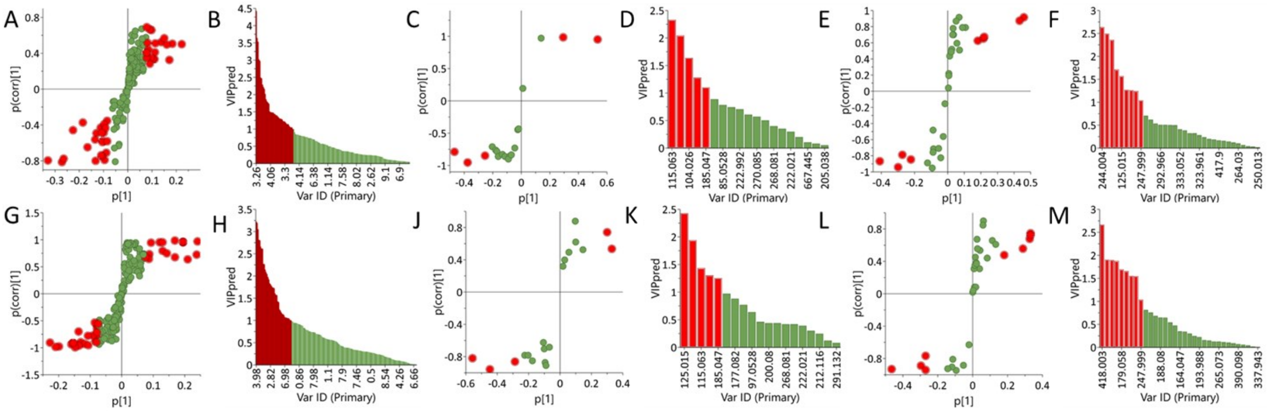

Figure S-1 OPLS-DA, S-plot and VIP plot of urine samples acquired by ^{1}H NMR spectra and LC-ESI-MS showing separation of control and treated tetracycline rats. Refer to method section for experimental details**. (A-B & G-H)** ^{1}H NMR spectra and **(C-F & J-M)** LC-ESI-MS. **(A & B)** control versus 62.5 mg/kg, **(G & H)** control verses 125mg/kg, **(C & D)** ESI+ control versus 62.5 mg/kg, **(J & K)** ESI+ control versus 125 mg/kg, **(E & F)** ESI- control versus 62.5 mg/kg and **(L & M)** ESI- control versus 125 mg/kg. Each point on the scores plot represents one sample. Control samples are represented by red circles (n=5), 62.5 mg/kg samples (n=5) are represented by light blue diamonds (n=5) and 125 mg/kg are represented by dark blue squares (n=5). See **Supplementary Information Table S1** for R^2X (cum), R^2Y cum) and Q^2 (cum) details.

Table S-1 PCA and OPLS-DA scores plot parameters. R^2X (cum) denotes the fraction of the variation of the X variables (the spectroscopic/spectrometric data) explained by the model, which represents goodness of fit, R^2Y denotes the fraction of the variation of the Y variables (the class information, e.g. control or treatment) that is explained by the model and Q^2 (cum) which estimates the predictability of the model.

Urine	NMR/ LC-ESI-MS	R^2X (cum)	R^2Y (cum)	Q^2 (cum)	CV-ANOVA	N
PCA control vs 62.5 mg/kg vs 125 mg/kg	NMR	0.612	-	0.397	-	15
OPLS-DA control vs 62.5 mg/kg	NMR	0.795	0.999	0.872	0.383	10
OPLS-DA control vs 125 mg/kg	NMR	0.695	0.996	0.914	0.007	10
OPLS-DA 62.5 mg/kg vs 125 mg/kg	NMR	0.696	0.975	0.920	0.005	10
PCA control vs 62.5 mg/kg vs 125 mg/kg	ESI +	0.984	-	0.554	-	15
OPLS-DA control vs 62.5 mg/kg	ESI +	0.803	0.982	0.957	0.001	10
OPLS-DA control vs 125 mg/kg	ESI +	0.775	0.980	0.961	0.001	10
PCA control vs 62.5 mg/kg vs 125 mg/kg	ESI -	0.925	-	0.672	-	15
OPLS-DA control vs 62.5 mg/kg	ESI -	0.972	0.999	0.97	0.022	10
OPLS-DA control vs 125 mg/kg	ESI -	0.873	0.915	0.864	0.021	10

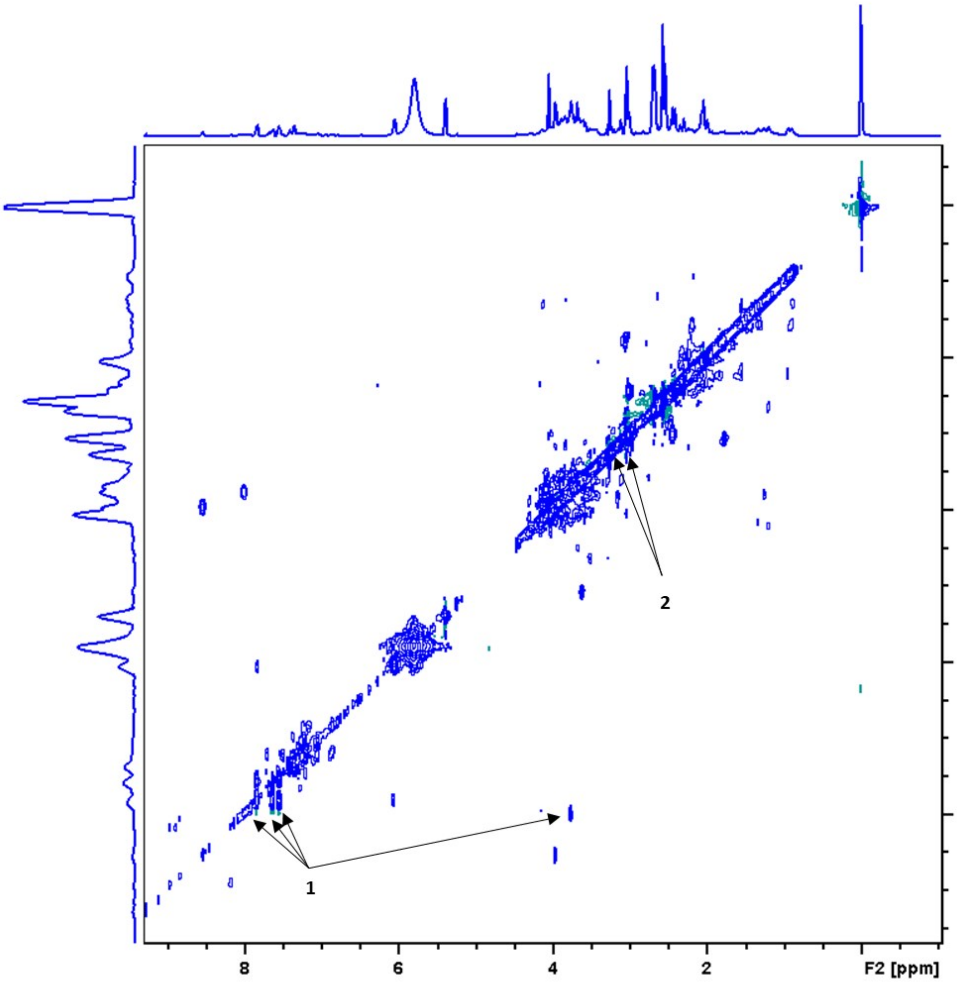

Figure S-2 Correlations observed in urinary of control rats 2D [1] H-[1] H TOCSY NMR spectrum. Lines indicate cross peaks that correspond to: 1. Hippurate 2. Taurine 3. Citrate

Table S-2 List of metabolites detected by ^{1}H NMR spectroscopy from urine of rats. Rats treated with either 62.5 mg/kg (n=5) or 125 mg/kg (n=5) tetracycline. Refer to methods for experimental details.

Metabolites	Tetracycline Treated Samples						
				62.5mg/kg		125mg/kg	
	Chemical shift (ppm)	Multiplices	P-value	Fold change	VIP	Fold change	VIP
Hippurate	7.84	d	0.016	-	-	3.209	2.275
Unidentified	7.66	s	0.013	-	-	1.388	0.013
Hippurate	7.62	t/d	0.005	-	-	2.387	0.005
Hippurate	7.56	t	0.060	-	-	2.589	2.621
Unidentified	7.46	d	0.046	0.594	1.762		
Unidentified	7.42	t	0.219	0.813	1.762	-	-
Unidentified	7.26	d	0.049	0.827	1.393	-	-
Unidentified	7.18	d	0.030	0.838	1.285	0.534	1.778
Unidentified	7.7	d	0.031	0.556	1.207	-	-
Unidentified	6.98	m	0.020	-	-	0.524	1.069

Unidentified	6.86	dd	0.030				1.738
Unidentified	6.82	s	0.008	0.728	1.297	-	-
Unidentified	6.74	dd	0.014	0.541	1.468	-	-
Unidentified	4.26	t	0.033	1.141	1.313	-	-
Unidentified	3.98	m	0.006	-	-	1.734	3.243
Hippurate	3.9	t	0.013	1.294	3.134	0.860	2.108
Unidentified	3.66	m	0.042	1.062	1.624	0.944	1.007
Unidentified	3.42	m	0.042	1.107	1.209	1.547	1.740
Unidentified	3.18	d	0.160	1.001	1.025	0.914	1.026
Unidentified	2.94	s	0.007	1.326	2.940	0.346	3.047
Unidentified	2.86	m	0.005	0.853	1.478	0.634	1.410
Unidentified	2.82	s/d	0.009	-	-	0.578	1.810
Citrate	2.66	d	0.015	-	-	2.224	1.935
Citrate	2.54	d	0.011	-	-	1.480	2.279
Unidentified	2.38	m	0.007	0.706	3.713	0.558	2.651
Unidentified	2.9	m	0.009	-	-	0.597	1.570
Unidentified	2.5	d	0.030	-	-	0.688	1.062
Lysine	1.98	m	0.016	0.924	1.377	0.868	1.206
Unidentified	1.94	m	0.003	0.715	3.613	0.508	2.801
Lysine	1.64	q	0.004	-	-	0.689	2.036
Unidentified	1.22	m	0.034	-	-	1.221	1.000
Unidentified	1.9	q	0.005	-	-	0.462	1.976

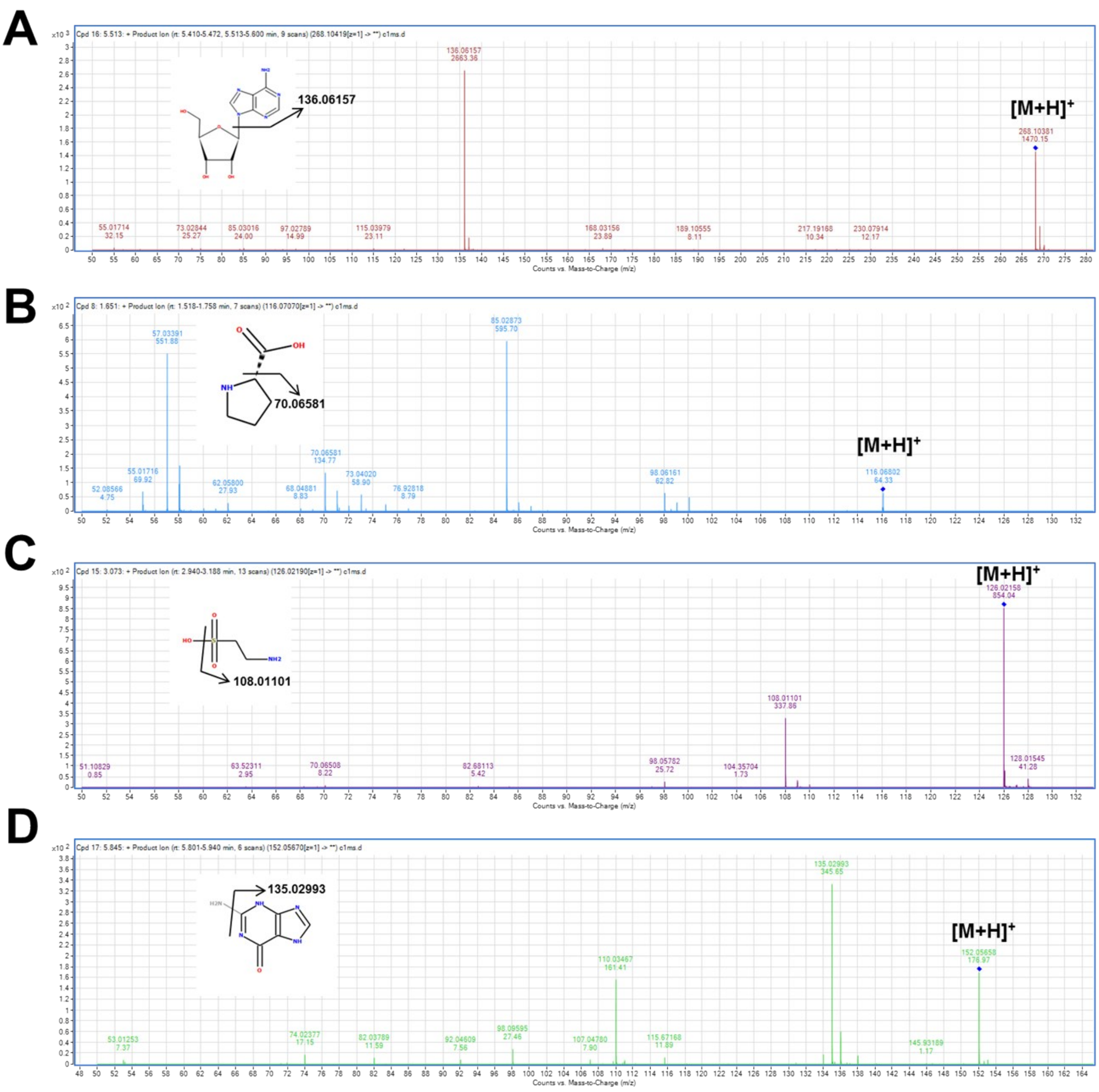

Figure S-3 Confirmation of the assignment of the molecules by MS/MS mass spectrometry for (**A**) Adenosine, (**B**) Proline, (**C**) Taurine and (**D**) Guanine. Experimental MS/MS spectrum for ion at *m/z* 268.104 (**A**), *m/z* 116.071 (**B**), *m/z* 126.022 (**C**) and *m/z* (**D**) 152.0658 in positive in mode $[M+H]^+$ at 20V of the peak at 5.513, 1.363 and 3.073 minutes retention time respectively.

Table S-3 List of metabolites detected by LC-ESI-MS/MS from urine of rats. Rats treated with either 62.5 mg/kg (n=5) or 125 mg/kg (n=5) tetracycline. Refer to methods for experimental details.

m/z	Mass	Identified Metabolite	Formula	Theoretical Mass	Δppm	RT (mins)	P-value	62.5 mg/kg Fold Change	VIP	125mg/kg Fold Change	VIP
116.071	115.06	Proline	$C_5H_9NO_2$	115.063	-4	1.808	0.026	4.684	3.66	2.966	2.95
269.088	268.08	Inosine	$C_{10}H_{12}N_4O_5$	268.081	-1	2.237	0.005	0.085	0.97	0.137	0.28
206.045	205.04	Xanthurenic acid	$C_{10}H_7NO_4$	205.038	0	0.831	0.379	1.130	0.3	1.235	0.37
268.104	267.10	Adenosine	$C_{10}H_{13}N_5O_4$	267.097	-1	5.913	-	-	-	-	-
152.057	151.05	Guanine	C_5H_5N5O	151.05	4	6.25	-	-	-	-	-
126.021	125.01	Taurine	$C_2H_7NO_3S$	125.014	2	3.279	-	-	-	-	-
